# Why digital health fails silently: a sociotechnical theory of health information technology–related risk

**DOI:** 10.3389/fdgth.2026.1785086

**Published:** 2026-05-15

**Authors:** Md Shafiqur Rahman Jabin

**Affiliations:** ^1^ Department of Medicine and Optometry, Linnaeus University, Kalmar, Sweden; ^2^ Faculty of Health and Social Care, University of Bradford, Bradford, United Kingdom

**Keywords:** complex adaptive system (CAS), human computer interaction, organisational learning (OL), patient safety, resilience engineering (RE)

## Abstract

**Introduction:**

Health information technology (HIT) is now integral to healthcare delivery, supporting clinical documentation, prescribing, diagnostics, and care coordination. Although these technologies offer substantial benefits, they have also introduced new patient safety risks that are often difficult to anticipate, detect, or manage. Many HIT-related safety problems arise not from isolated technical failures or individual mistakes, but from complex interactions between digital systems, clinical work practices, organisational structures, and governance arrangements. Traditional patient safety models that focus on discrete errors or linear causality are therefore insufficient for explaining how digital risks emerge and persist in practice.

**Methods/theoretical approach:**

This article develops a sociotechnical theory of HIT-related risk grounded in patient safety science and sociotechnical systems theory. The theory is informed by empirical insights from incident-based research on HIT-related safety problems and synthesises evidence from real-world incident narratives. It adopts a conceptual, theory-building approach informed by purposive, iterative engagement with the relevant literature on health IT safety, sociotechnical systems, and resilience-oriented patient safety frameworks. Rather than analysing a single dataset, the paper identifies recurring mechanisms through which digital risks arise, remain hidden, propagate across contexts, and become recoverable or not.

**Results/theoretical propositions:**

The proposed theory conceptualises HIT-related risk as a dynamic process involving four interrelated mechanisms: risk emergence, risk concealment, risk propagation, and recoverability. Risks emerge through misalignments between system design, configuration, and clinical workflows; they are concealed by automation, information fragmentation, and adaptive workarounds; they propagate through tightly coupled digital infrastructures and shared dependencies; and their recoverability depends on organisational capacity for detection, escalation, and learning. Together, these mechanisms explain why HIT-related incidents may affect multiple patients or services, why attribution to individual error is misleading, and why safety problems may persist despite corrective efforts.

**Discussion/implications:**

By reframing HIT-related incidents as manifestations of system-level vulnerabilities rather than isolated failures, this sociotechnical theory provides a coherent explanatory framework for understanding digital patient safety. It highlights how risks can evolve silently within routine practice, vary in visibility and scale, and emphasises the importance of organisational learning, governance, and resilience in managing digital safety risks.

## Introduction: the paradox of safety in digital healthcare

Health information technology (HIT) is now embedded across most clinical processes, including documentation, prescribing, diagnostic workflows, and cross-organisational information exchange (1). This expansion has delivered substantial benefits, but it has also introduced new categories of safety risk that are often difficult to anticipate, detect, and manage. Digital technologies can alter established work practices, redistribute cognitive workload across clinicians (including doctors, nurses, and allied health professionals), and increase coupling and interdependence between system components (2, 3). These changes may improve efficiency and standardisation, yet they can also obscure failure mechanisms, delay detection, and complicate recovery when problems occur (1, 4). For example, digital health–related incidents have included incorrect patient selection in electronic records, data mismatches leading to delayed or inappropriate treatment, and emerging concerns about AI-supported diagnostic systems producing misleading outputs that require careful human oversight (5, 6).

A central challenge is that many clinically significant HIT-related failures become visible only after implementation, when digital systems are integrated into the variability of real-world care. Evidence from incident-report analyses demonstrates that HIT-related problems frequently arise from interactions among system design, configuration, workflow, and human adaptation, rather than from isolated technical faults (1, 7). Incident-based studies, spanning patient identification/detail issues and multi-patient events, i.e., illustrate how risks can propagate across patients, settings, and time, and how “small” informational problems may generate disproportionate downstream consequences (8, 9).

These challenges can be more fully understood by drawing together insights from complexity science and sociotechnical systems theory, which offer complementary ways of conceptualising how healthcare systems function. Complexity theory characterises healthcare as a dynamic system in which outcomes emerge from non-linear interactions, feedback loops, and evolving conditions over time (10, 11). In such systems, cause–and–effect relationships are not always predictable, and small changes, such as the introduction of new digital technologies, can produce disproportionate and sometimes unintended consequences.

Sociotechnical perspectives extend this view by emphasising that these dynamics arise from the interdependence between technological artefacts, human actors, workflows, and organisational structures (12, 13) (Sittig and Singh, 2010; Harrison et al., 2007). Rather than treating technology as separate from practice, sociotechnical theory highlights how digital systems actively reshape work processes, redistribute cognitive tasks, and introduce new dependencies across system components. The introduction or modification of digital technologies can therefore be understood as a form of system perturbation, altering established relationships and creating new conditions under which risks may emerge, interact, and evolve (5, 14).

Within this combined framing, safety is not simply the absence of error but an emergent property of the system as a whole. This perspective aligns with developments in patient safety science, including the distinction between Safety-I (focused on preventing failures), Safety-II (focused on understanding how systems succeed under varying conditions), and more recent extensions that emphasise system adaptability and learning (15–17).

Recent safety investigations and policy work reinforce the growing importance of “digital safety” as a patient safety priority. The WHO's Global Patient Safety Action Plan explicitly emphasises system-level learning and the need to address emerging risks across modern healthcare delivery, including those shaped by technology (18). In the UK, a 2025 thematic review by the Health Services Safety Investigations Body (HSSIB) synthesised multiple investigations into electronic patient record systems, highlighting recurring safety issues related to implementation, workflow fit, and organisational capacity to detect and respond to hazards (19). Alongside this, recent patient safety scholarship has argued that digital systems create distinctive safety dynamics that cannot be addressed using traditional approaches alone (2).

A range of theoretical perspectives have been used to understand the implementation and impact of digital technologies in healthcare, but each provides only a partial account of the safety challenges observed in practice. Technology adoption models, such as the Technology Acceptance Model (TAM) and the Unified Theory of Acceptance and Use of Technology (UTAUT), have been widely applied to explain user uptake and acceptance of digital systems, emphasising perceived usefulness, ease of use, and behavioural intention (20, 21). Similarly, information systems success models, such as the DeLone and McLean framework, focus on system quality, information quality, and user satisfaction as determinants of successful implementation (22).

While these approaches offer valuable insights into adoption and performance, they are less well-suited to explaining how risks emerge dynamically within complex, real-world clinical environments. Sociomaterial perspectives and implementation theories, such as Normalisation Process Theory, address this challenge by examining how technologies and social practices become intertwined in routine work (23). However, even these approaches tend to focus on integration and use, rather than on how risks remain hidden, propagate across systems, or become visible only through downstream consequences.

In the patient safety domain, models such as the sociotechnical framework proposed by Sittig and Singh (2010) and broader systems-based approaches have advanced understanding of how technology-related risks arise from interactions across multiple system dimensions (24). However, these models do not fully explain the temporal and dynamic characteristics of digital risk, including how risks can remain concealed, scale across patients or services, and challenge detection and recovery in practice. Taken together, these limitations point to the need for a more integrative theoretical account that explicitly addresses how digital health risks emerge, evolve, and persist within digitally mediated care systems.

Despite increasing attention, there remains a gap between (i) documenting HIT-related problems and (ii) offering an explanatory theory of *why* these risks emerge and *how* they become concealed, amplified, or recoverable in everyday practice. This paper addresses that gap by developing a sociotechnical theory of HIT-related risk grounded in patient safety science and informed by incident evidence from real-world clinical settings.

To address this gap, this paper develops a sociotechnical theory of HIT-related risk that explains how risks emerge, remain concealed, propagate, and become recoverable in practice. The paper is structured to progressively build this argument. First, it outlines key sociotechnical and safety science foundations relevant to digital healthcare. It then examines why HIT-related risks are often difficult to detect in routine practice. Building on these insights, the paper presents the proposed theoretical framework, followed by a discussion of its implications for research, practice, and policy. This structure is intended to guide the reader from foundational concepts to an integrated theoretical explanation of digital safety risk.

## Conceptual foundations: sociotechnical safety in complex digital systems

This paper is anchored in the view that healthcare is a digitally mediated care system in which outcomes emerge from interactions among people, technologies, tasks, organisational arrangements, and external constraints. This section lays the conceptual groundwork necessary for developing the proposed sociotechnical theory of HIT-related risk.

Sociotechnical perspectives do not treat technology as an isolated “tool” but as an active element that shapes work, cognition, coordination, and the distribution of responsibility and control. As a result, HIT-related incidents are best understood not as discrete failures but as manifestations of system-level vulnerabilities that arise from design decisions, implementation conditions, workarounds, and interdependencies.

Two conceptual foundations are especially relevant for explaining HIT-related risk. First, system coupling and escalation: digital infrastructures often increase coupling across workflows and units, enabling local problems (e.g., data integrity issues, identity mismatches, configuration errors) to propagate across multiple patients or services (8, 9). The analysis of incidents affecting the management of multiple patients provides empirical grounding for this principle, showing how shared digital dependencies can amplify impact beyond single cases (8, 9).

Second, process and temporality: HIT-related hazards frequently unfold across time, with delayed detection and downstream consequences that become visible only at later workflow stages. The SEIPS 3.0 model explicitly extends sociotechnical thinking by emphasising the *patient journey over space and time*, offering a valuable lens for examining when and where digital failures emerge and how they propagate (25). This temporal framing aligns closely with incident evidence from the work on patient details-related challenges, where problems are often introduced at one step (e.g., data entry, system selection, identity handling) but realised later through medication, imaging, referral, or communication processes (4, 8, 9).

Finally, contemporary patient safety discourse increasingly distinguishes between preventing known failures and strengthening the system's ability to respond, recover, and learn under variability. This resilience-oriented view is particularly important in digital healthcare, where failures cannot be fully eliminated, and safety increasingly depends on visibility, monitoring, recovery pathways, and governance of change (26). Recent Frontiers work on digital resilience at the system level further supports the relevance of resilience concepts for understanding and managing risk in digitally mediated healthcare environments.

Together, these foundations motivate the core aim of this article: to develop a sociotechnical theory explaining how HIT-related risks emerge, remain hidden, propagate, and become recoverable (or not) in routine clinical practice (13, 17).

## Why HIT-related risks often remain silent

Building on these foundations, this section examines a key problem that the theory seeks to explain: why HIT-related risks are often difficult to detect in practice. However, this difficulty is not absolute. As patterns of failure become better understood through experience, incident reporting, and organisational learning, some risks become more predictable and can be more effectively anticipated and managed in subsequent implementations (10, 15, 27).

A defining characteristic of many HIT-related safety problems is that they remain silent within routine clinical practice. Unlike traditional clinical hazards, which may be directly observable at the point of care, digital risks often emerge indirectly, unfold over time, and become apparent only through their downstream consequences. Understanding the silent nature of risk is central to developing a sociotechnical theory of digital safety.

While these mechanisms are described here to explain why risks are difficult to detect in practice, they are later synthesised into a broader theoretical framework.

### The silent nature of risk and the opacity of digital systems

Digital systems frequently mediate clinical work in ways that reduce direct visibility of system state and process integrity. Automation, abstraction, and layered interfaces can mask underlying system behaviour, making it difficult for clinicians to recognise when information is incomplete, delayed, mismatched, or erroneous (5, 13, 28). As a result, problems may remain latent until they intersect with clinical decision-making or patient care activities, at which point recovery may be constrained by time pressure or organisational dependencies.

Incident-based evidence demonstrates that HIT-related problems are often detected indirectly, for example, through unexpected clinical outcomes, discrepancies across systems, or the need for manual reconciliation, rather than through explicit system alerts. The analyses of patient details–related incidents illustrate how identity mismatches, selection errors, or data integrity problems may persist unnoticed across multiple workflow stages, only becoming visible when care is delayed, duplicated, or misdirected (29, 30). In such cases, the digital system does not simply fail; rather, it fails silently. While some system failures are immediate and highly visible, many HIT-related risks develop in less observable ways, becoming apparent only through their downstream effects, which is the primary focus of this analysis.

This silent character of risk reflects a broader shift in how safety is manifested in digitally mediated environments. Rather than presenting as immediate and observable failures, risks are often embedded within routine processes and only become apparent when system conditions align in particular ways (15–17). This aligns with sociotechnical and resilience perspectives, which emphasise that safety and failure emerge from the same underlying system dynamics and may remain latent until triggered by specific interactions.

The opacity of digital systems is further reinforced by increasing system complexity and interoperability, where multiple interconnected platforms exchange and transform data across organisational boundaries. In such environments, the underlying logic of information processing is often inaccessible to end users, limiting their ability to verify data provenance or system behaviour (5, 12). This lack of transparency has been identified as a key contributor to technology-related safety risks, particularly in systems that rely heavily on automation and integration.

### Redistribution of cognitive work and responsibility

Digitisation does not eliminate cognitive work but redistributes it across people, systems, and organisational structures. Tasks previously performed explicitly by clinicians, such as cross-checking information, tracking patient identity, or coordinating across services, may be partially delegated to digital systems (31, 32). While this redistribution can increase efficiency, it can also create new dependencies and assumptions about system reliability. When these assumptions are violated, clinicians may lack both the information and the authority needed to detect or correct problems in a timely manner (31, 33).

This redistribution also affects how responsibility is perceived and enacted. When information is produced or transformed by multiple interconnected systems, it may be unclear who is responsible for verifying its accuracy or responding to anomalies (8, 34, 35). Incident narratives frequently reflect this ambiguity, with reporters uncertain whether problems originate in user actions, system configuration, data flows, or organisational processes. Such ambiguity contributes to delayed detection and fragmented recovery efforts.

This redistribution of cognitive work also reshapes how clinicians engage with information, often requiring them to manage multiple digital interfaces and reconcile fragmented data sources (25, 33). As a result, cognitive load may increase rather than decrease, particularly in complex or high-pressure environments. Studies have shown that such conditions can contribute to oversight, misinterpretation, or delayed recognition of anomalies, further reinforcing the conditions under which risks remain undetected.

### Temporal displacement and downstream effects

A further challenge lies in the temporal characteristics of HIT-related risk. Problems introduced at one point in the workflow, such as during data entry, system configuration, or system updates, may not manifest until much later, when information is retrieved, reused, or integrated into new clinical contexts. This temporal displacement complicates causal attribution and can obscure the relationship between an initiating condition and its eventual consequences (25, 36).

The work on incidents affecting multiple patients provides clear examples of this dynamic, showing how configuration errors or shared digital dependencies can propagate across time and scale, affecting many patients before the underlying issue is recognised (1, 8, 9, 37). Such incidents challenge conventional notions of “near misses” and “single-patient events” and underscore the importance of examining how digital risks accumulate and propagate within tightly coupled systems (1, 38).

Temporal displacement also means that responsibility for risk detection is often disconnected from the point at which the risk is introduced. Clinicians encountering downstream consequences may lack the contextual information needed to identify the problem's origin, making recovery more difficult (25, 36). This highlights the importance of considering time as a critical dimension of sociotechnical risk, particularly in systems where processes span multiple stages, actors, and organisational settings.

### Normalisation, workarounds, and masked risks

Finally, the adaptive capacity of healthcare professionals can paradoxically contribute to the silent nature of HIT-related risk. Clinicians routinely develop workarounds to cope with system limitations, usability issues, or workflow mismatches. While these adaptations may enable care to continue, they can also mask underlying system vulnerabilities and reduce organisational awareness of risk. Over time, such workarounds may become normalised, further embedding risk within everyday practice (12, 15, 17).

Incident reports often capture moments when these adaptive strategies fail, either when a workaround no longer suffices or when multiple vulnerabilities align. From a sociotechnical perspective, these moments should not be interpreted as isolated breakdowns, but as signals of deeper system-level tensions between technology, work practices, and organisational constraints (1, 39). These observations collectively point to the need for a more integrated explanation of how such mechanisms interact, which is developed in the following section.

Over time, the normalisation of workarounds can lead to a gradual erosion of system safeguards, as informal practices replace formal processes without corresponding visibility at an organisational level. This creates a form of “drift” in system performance, where deviations from intended use accumulate without being recognised as safety concerns (15–17). Such dynamics are central to resilience-based safety theory, which highlights how adaptive behaviour can both support and undermine safety depending on system conditions.

## A sociotechnical theory of hit-related risk

Building on the preceding analysis, this section presents a sociotechnical theory of HIT-related risk that explains how safety problems emerge, remain hidden, propagate, and become recoverable within digital healthcare systems. In this view, HIT-related incidents are not best explained as isolated technical malfunctions or individual user errors; rather, they arise through interactions between system design, configuration, workflow conditions, organisational governance, and the adaptive strategies clinicians develop to keep care moving. This theory, therefore, explains risk through four interlocking processes: emergence, concealment, propagation, and recoverability. Rather than repeating prior observations, this section integrates these mechanisms into a coherent explanatory framework that accounts for how risks develop and interact across system levels and the extent to which they may impact patients, services, or entire systems. In this way, the framework incorporates both the mechanisms of risk generation and the scale and consequences of their propagation in practice.

### Risk emergence

Risk emerges when digital systems introduce new constraints, assumptions, and dependencies into clinical work. These risks may originate in the technical artefact (e.g., interface behaviour, data models, integration logic), but they become clinically consequential only when embedded into workflows, roles, and organisational routines (1, 40). Sociotechnical models of HIT emphasise that safety should be examined across interacting dimensions, i.e., technology, people, workflow, organisational policies, and external pressures, because hazards typically arise from misalignments between these elements rather than from any single component alone (13, 41, 42).

Incident-based work provides a consistent empirical grounding for this point: patient details–related risks (e.g., identity selection, mismatched records, data integrity issues) frequently reflect design–workflow misfits rather than “simple mistakes,” and multi-patient incidents illustrate how shared digital dependencies can create failure modes that are qualitatively different from traditional single-patient errors (1, 7, 37, 43, 44).

Recent evidence from large-scale EHR implementation experiences also reinforces that local configuration decisions and implementation conditions can introduce workflow friction and safety hazards even when the underlying platform is stable (40). From this perspective, such risks should not be viewed as exceptional events but as inherent and often unavoidable features of complex sociotechnical systems, arising naturally from interactions between system components and evolving conditions over time (16, 17, 27).

### Risk concealment

A distinctive feature of digital risk is that it can remain hidden. HIT hazards are often *latent* because digital systems reduce visibility of their internal state and because errors may be expressed as *information problems* (wrong, missing, delayed, fragmented, or ambiguous data) rather than immediately observable clinical events. This concealment is intensified by automation, abstraction, and distributed information flows across multiple systems and services (6).

Concealment is also socially produced. Clinicians adapt through workarounds, informal checks, and local fixes. These strategies can be effective in the short term but may mask systemic vulnerabilities, delay organisational learning, and normalise degraded modes of operation (6). Patient- and clinician-facing accounts of unintended consequences in digital health repeatedly show that increased burden, loss of continuity, and fragmented information can persist without being recognised as “safety events” until a tipping point is reached (6, 45, 46).

However, concealment is not universal. Some risks become immediately visible following system implementation, particularly in cases such as incorrect patient selection, medication errors, data conversion failures, or misinterpretation of AI-supported outputs. These more observable failures coexist with less visible risks, highlighting that detectability varies depending on system design, context, and stage of use (5, 12).

### Risk propagation

Propagation refers to how HIT-related problems scale across time, people, and patients. Digital systems can increase coupling between components and workflows, enabling local anomalies to cascade through shared infrastructure (e.g., configuration changes, template updates, interoperability interfaces, shared patient identifiers). This is particularly salient for incidents that affect multiple patients or services, where a single upstream condition can generate repeated downstream consequences before it is detected (8, 9).

From a systems perspective, this aligns with the logic of complex, tightly coupled systems: when dependencies are high and processes are time-sensitive, small disturbances can propagate quickly and unpredictably. In contemporary healthcare, this propagation is not only technical but organisational, spanning governance structures, reporting systems, and vendor–provider relationships (11, 47). Notably, a 2025 HSSIB thematic review synthesising investigations that considered EPR systems highlights recurring safety issues that relate to implementation, workflow fit, and organisations' ability to detect and respond to hazards at scale (47). Importantly, the scale of impact can vary significantly, ranging from single-patient effects to system-wide consequences affecting multiple patients, services, or organisational units, particularly in highly interconnected digital environments (8, 9, 27, 30).

### Recoverability and resilience

Recoverability concerns whether, how, and how quickly a system can detect, contain, and correct problems once they occur. In digitally mediated care, recovery depends on the availability of effective signals (that something is wrong), clear responsibility and authority (to act), and workable pathways (downtime procedures, escalation routes, verification mechanisms). Resilience-oriented perspectives emphasise that safety is not only the absence of failure but also the capacity to respond and adapt under variability (26).

Operational guidance for strengthening recoverability and safe EHR use is increasingly being formalised. The 2025 revision of the SAFER Guides (Safety Assurance Factors for EHR Resilience) reflects an ongoing effort to translate EHR safety concerns into actionable organisational practices, including contingency planning, monitoring, and governance (48). Quantitative and qualitative studies continue to show wide variation in EHR safety practices and underline that resilience is not a property of the software alone, but of the sociotechnical system that surrounds it (49).

### Putting the theory together

This sociotechnical theory, therefore, explains HIT-related risk as a *dynamic trajectory*:

1. **Risk emerges** through design, configuration, integration, and workflow embedding.
2. **Risk is concealed** by opacity, distributed information flows, and adaptive workarounds.
3. **Risk propagates** through coupling across systems, services, and patients.
4. **Risk becomes recoverable (or not)** depending on organisational capacity for detection, escalation, and learning.

Crucially, these processes are interdependent: concealment increases propagation; propagation tests recoverability; weak recoverability encourages further workarounds, which can deepen concealment. This helps explain why many HIT safety problems persist across implementations and why improvement efforts must address not only software defects but also organisational governance, workflow design, and learning systems (8, 9).

**Figure 1:**
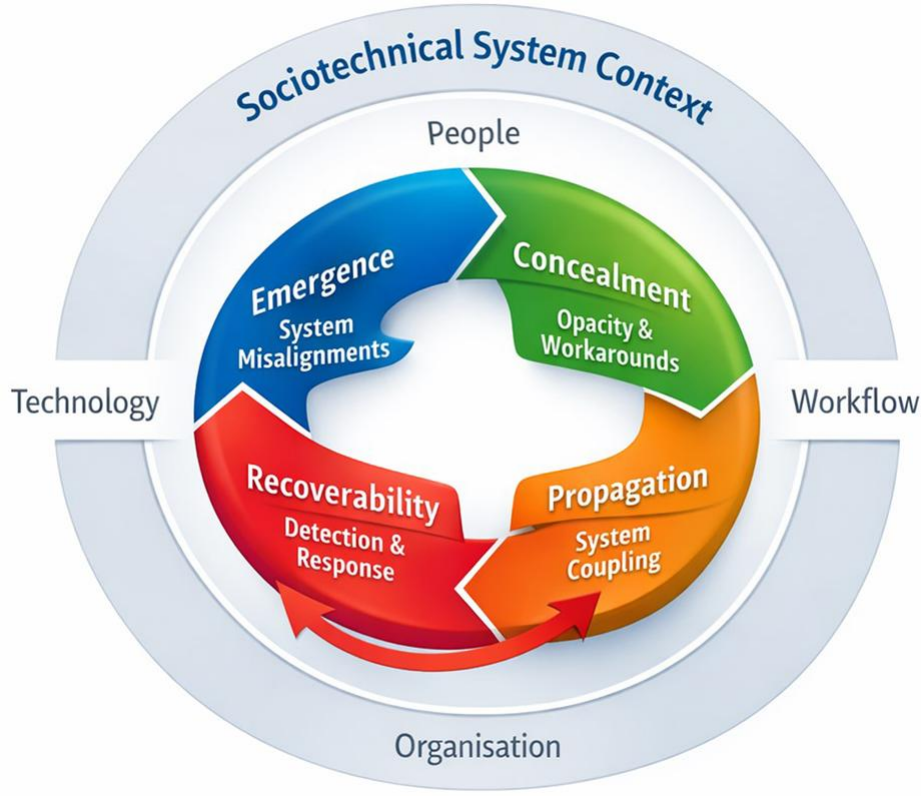
A sociotechnical model of HIT-related risk as a dynamic and often silent process. Figure 1 illustrates how the proposed theory can be used to understand the development of HIT-related risk in practice. Rather than representing a linear sequence, the model depicts a dynamic cycle in which risks emerge from misalignments between system components, remain concealed through system opacity and adaptive practices, propagate across interconnected workflows and infrastructures, and become recoverable (or not) depending on organisational capacity for detection and response. Crucially, these mechanisms are interconnected: concealment can enable further propagation, propagation increases the likelihood of delayed detection, and limitations in recoverability can reinforce conditions that allow risks to persist. This cyclical and feedback-driven structure reflects the behaviour of complex sociotechnical systems and provides a practical framework for analysing how digital safety problems evolve over time in real-world settings.

This theory, therefore, explains risk through four interlocking processes: emergence, concealment, propagation, and recoverability. The dynamic and interdependent relationships among these mechanisms are illustrated in Figure 1, which presents the theory as a cyclical process embedded within a broader sociotechnical system.

## Reframing “error” and responsibility in digital healthcare

Traditional patient safety frameworks have largely conceptualised incidents as discrete errors attributable to individual actions, protocol deviations, or procedural failures. While such models have proven useful for understanding certain categories of clinical harm, they are increasingly inadequate for explaining safety problems in digitally mediated healthcare (13). HIT-related incidents frequently arise in contexts where clinicians act reasonably within the constraints of complex systems, yet adverse outcomes still occur. A sociotechnical perspective, therefore, requires a fundamental reframing of how error and responsibility are understood in digital healthcare.

### Limits of individual error models in HIT-related incidents

In many incident reports involving HIT, the immediate description of what went wrong may reference a user action, such as selecting the wrong patient, entering incorrect information, or failing to notice an alert. However, closer examination often reveals that these actions occurred within system conditions that shaped, constrained, or normalised such behaviour (5, 31). Interface design, default settings, fragmented information displays, time pressure, and workflow misalignment can all influence how clinicians interact with digital systems, rendering simplistic attributions of “user error” analytically misleading.

Analyses of patient details–related incidents provide concrete illustrations of this problem. Events initially framed as selection or documentation errors frequently reflect deeper sociotechnical issues, such as identity management across systems, interface ambiguity, or the need to manage multiple patients simultaneously within tightly coupled digital workflows (8, 9, 50, 51). In these cases, focusing on individual performance obscures the structural conditions that make such incidents likely to recur.

Recent patient safety scholarship has increasingly challenged error-centric explanations in digital contexts, arguing that they underestimate the role of system design and overestimate the capacity of individuals to compensate for poorly aligned technologies (25, 52, 53).

### Redistribution of responsibility in sociotechnical systems

Digitisation redistributes responsibility across human and technical actors. Decisions about information accuracy, timing, and relevance are increasingly mediated by algorithms, system configurations, and interoperability rules that are often opaque to end users (1, 13). As a result, responsibility for safety becomes diffused across clinicians, system designers, vendors, implementers, and organisational governance structures. This diffusion complicates accountability and can create gaps in oversight, particularly when responsibilities for monitoring, escalation, and remediation are not clearly defined (13).

Incident narratives frequently reflect this ambiguity. Reporters may express uncertainty about whether problems originated in clinical practice, system configuration, data flows between systems, or organisational decisions made long before the incident occurred. The work on incidents affecting multiple patients highlights how shared digital dependencies can create situations in which no single individual has full visibility or control over the conditions that give rise to harm (8, 29). In such contexts, attributing responsibility to frontline staff is not only analytically inadequate but may also undermine learning by discouraging reporting and masking systemic vulnerabilities. In practice, this may involve implementing shared responsibility models that integrate clinical, technical, and organisational perspectives, such as multidisciplinary incident reviews, clearer escalation pathways, and system-level monitoring mechanisms that support early detection and coordinated response to emerging risks (13, 16, 17).

### From blame to system accountability

A sociotechnical reframing shifts the focus from individual blame to system accountability. This does not imply the absence of professional responsibility, but rather a recognition that safety outcomes emerge from interactions across multiple system levels. Contemporary safety science emphasises that learning is more likely to occur when organisations examine how system conditions shape behaviour, rather than asking why individuals failed to comply with expectations that may be misaligned with real-world constraints (15, 17, 54).

In digital healthcare, this shift is particularly important because many HIT-related risks arise from design and implementation decisions that are temporally and organisationally distant from frontline care. Configuration changes, software updates, and integration choices may introduce new hazards without corresponding mechanisms for detection or feedback. Holding individuals responsible for managing these latent risks places unrealistic demands on clinicians and risks perpetuating cycles of underreporting and superficial fixes.

### Implications for learning and improvement

Reframing error and responsibility have direct implications for how organisations learn from HIT-related incidents. Incident reporting systems that implicitly frame events as individual mistakes may fail to capture sociotechnical contributing factors and discourage candid reporting. In contrast, approaches that invite reflection on system design, workflow integration, and organisational context are better suited to identifying recurrent patterns and informing preventive strategies (35, 52).

Recent guidance on digital safety governance, including updates to the SAFER Guides, reflects growing recognition of the need for shared responsibility and organisational accountability in managing EHR-related risks. These frameworks emphasise governance, monitoring, and continuous learning as essential complements to technical controls and user training (48, 55). From a sociotechnical perspective, such developments represent a shift towards more mature models of responsibility that align with the realities of complex digital healthcare systems.

## Discussion

This discussion interprets the proposed sociotechnical theory of HIT-related risk and situates it within the broader patient safety and digital health literature. Rather than presenting implications as isolated observations, this section explains what the theory reveals about the nature of digital risk, why existing approaches are insufficient, and how this work extends current sociotechnical and safety perspectives. The discussion is structured around key domains in which the theory is relevant: implications for patient safety research, clinical practice and safety management, and policy- and system-level governance. Across these areas, we examine how the concepts of emergence, concealment, propagation, and recoverability provide a coherent framework for understanding and addressing risks in digitally mediated healthcare systems.

The value of this theory lies in its ability to explain not only why digital risks occur but also how they develop, remain undetected, and evolve across complex healthcare systems. Existing approaches, including sociotechnical models and safety frameworks, have provided important insights into the role of system interactions and organisational context (13, 16, 17, 28, 48). However, they tend to focus on static representations of system components or discrete incidents, rather than on capturing the dynamic, temporal processes through which risks emerge, become concealed, and propagate across patients and settings. By conceptualising risk as a trajectory shaped by interacting mechanisms, this theory extends these approaches and provides a more integrative explanation of digital safety. The inclusion of feedback loops, as illustrated in Figure 1, further highlights how risks can evolve over time, reinforcing the need to move beyond linear models of causality. This perspective offers a more realistic account of digitally mediated care and helps explain why safety problems may persist despite corrective efforts.

### Implications for patient safety research

The sociotechnical theory articulated in this paper has several implications for how patient safety research should be conceptualised, designed, and conducted in digital healthcare environments. Rather than treating HIT as an inert tool whose effects can be evaluated in isolation, safety research must recognise HIT as an active agent within complex work systems. This shift has consequences for theory, methodology, and empirical practice.

#### Expanding the unit of analysis

Traditional patient safety studies often focus on *proximal causes* of incidents, such as individual actions or specific technical faults. In contrast, sociotechnical theory highlights that HIT-related risk emerges from interactions across multiple system layers from user interfaces to organisational policies, vendor practices, and regulatory environments. Consequently, safety research should expand its unit of analysis to include not only the clinician–technology interface but also the broader system relationships in which digital tools are embedded (1, 42).

Recent work underscores the value of systems approaches in complex digital environments. For example, research syntheses argue that safety improvement depends on understanding how information flows, organisational routines, and technological artefacts co-evolve over time (16, 25). These perspectives align with our theory and suggest that incident analysis must be complemented by methods that capture multi-level interactions and temporal dynamics (1).

#### Rethinking measurement and data sources

Measurement in HIT safety research has often emphasised *counts* of errors or adverse events as performance indicators. However, the sociotechnical lens suggests that counting alone cannot reveal *why* incidents occur or *how* they propagate. Instead, research should prioritise rich, qualitative data sources, such as narrative incident reports, structured interviews, and process observations, to reveal latent mechanisms and contextual conditions (1, 10, 11).

Several methodological cross-disciplinary reviews have advocated for triangulating qualitative and quantitative methods to capture complexity in digital health safety (32, 56). Such approaches allow researchers to identify not just what went wrong, but how organisational context, workflow structure, and technology configuration converge to create risk. This aligns with an incident-driven approach, which has demonstrated the value of narrative synthesis for identifying recurring risk patterns that would be silent to purely statistical surveillance (1).

#### Longitudinal and adaptive research designs

The theory also implies that HIT-related risks are not static but evolve as systems change. Digital platforms are routinely updated, configured, and integrated with other systems, meaning that safety characteristics at one point in time can differ significantly from those at another. Longitudinal research designs, including repeated incident analysis, time-series evaluation of safety indicators, and adaptive case studies are, therefore, essential for understanding how risks unfold across the lifecycle of digital systems (1, 11, 57).

Emerging work on *digital safety trajectories* suggests that risks often re-emerge after system upgrades, organisational restructures, or policy changes, underscoring the need for continuing safety surveillance rather than one-off evaluations (16, 58). Integrating such longitudinal perspectives with sociotechnical analysis can help researchers detect *emergent hazards* early and inform more resilient system design (16, 59).

#### Integrating resilience and safety-II perspectives

Safety science has increasingly shifted toward resilience-oriented frameworks (Safety-II), which emphasise systems' capacity to succeed under varying conditions rather than merely preventing failures. A sociotechnical theory of HIT-related risk naturally complements this shift by focusing on how work systems adapt, how risks are concealed and revealed, and how recovery occurs in practice (16, 59).

Recent digital health safety work calls for integrating Safety-II principles, such as variability as a source of insight and success, into HIT evaluations (16, 59). This perspective encourages researchers to study not only incidents that *fail* but also instances in which systems and practitioners successfully manage variability, offering a richer understanding of safe practice (16, 17, 59).

#### Methodological implications

Finally, the theory highlights the need for methodological pluralism in patient safety research. Complex digital systems demand mixed methods, including qualitative incident analysis, process mining, simulation, ethnography, and systems modelling. These methods contribute complementary insights into how HIT impacts work, cognition, and outcomes. Moreover, reporting guidelines for HIT safety studies should emphasise transparency in contextual description, system configuration details, and organisational factors because these often determine where risk arises (1, 17).

By adopting these expanded methodological toolkits, patient safety research can generate deeper, more actionable knowledge about digital risks, support more effective interventions, and inform policy and governance frameworks that align with how digital healthcare actually functions in practice (1, 17, 60).

### Implications for clinical practice, safety management, and governance

The sociotechnical theory developed in this article has practical implications for how healthcare organisations manage safety in digitally mediated care. Rather than focusing primarily on preventing individual errors, the findings suggest that safer digital healthcare depends on strengthening organisational capacity to detect emerging hazards, contain their effects, and learn from system-level vulnerabilities (28, 55).

In this paper, the concept of “learning systems” refers to the organisational capacity to continuously detect, interpret, and respond to safety-relevant signals arising from routine practice, particularly in digitally mediated environments. This usage overlaps with, but is not identical to, the concept of Learning Health Systems, which emphasises the systematic integration of data, evidence, and practice improvement at scale (56). Here, the focus is more specifically on how organisations learn from sociotechnical interactions, including incident reports, near misses, and everyday adaptations, to identify latent system vulnerabilities. This perspective also aligns with broader notions of learning organisations and systems thinking, but emphasises the dynamic, feedback-driven processes through which digital risks are recognised, escalated, and addressed in practice (11).

At the level of clinical practice, increasing the visibility of digital risk is critical. Because many HIT-related problems remain latent until downstream effects occur, organisations should support mechanisms that enable clinicians to recognise information anomalies and escalate concerns without relying solely on individual vigilance. This includes valuing incident reports and informal safety signals as early indicators of system drift, particularly during periods of change such as system upgrades or workflow redesign (15, 17).

For safety management, the theory highlights the importance of *recoverability* as a core safety property. Effective recovery depends on timely detection, clear responsibility for action, and workable contingency arrangements that allow care to continue when digital systems fail. Organisational guidance such as the SAFER Guides reflects this shift by emphasising governance, monitoring, and continuous self-assessment as essential components of EHR safety, rather than treating safety as a function of software reliability alone (28, 48).

Finally, the theory underscores that governance of digital change is itself a patient safety function. Risks are often introduced through local configuration decisions, system integrations, and ongoing modifications, rather than through initial system design. This implies the need for sustained oversight throughout the lifecycle of digital systems, including post-implementation monitoring and multidisciplinary review of incidents involving sociotechnical interactions. Strengthening learning systems to support such review can help organisations move beyond reactive fixes and address recurrent patterns of risk in digital healthcare (42, 47).

### Policy and system-level implications

The sociotechnical theory of HIT-related risk also has implications beyond individual organisations, particularly for policy, regulation, and system-level learning. As digital health infrastructures increasingly span organisational and sectoral boundaries, risks associated with design, configuration, and interoperability cannot be managed solely at the local level (1, 42, 61). Effective oversight, therefore, requires coordinated approaches that recognise HIT-related safety as a shared responsibility across providers, vendors, regulators, and national safety bodies.

From a policy perspective, the theory highlights the limitations of regulatory approaches that focus primarily on pre-deployment assurance or compliance with technical standards. Many clinically significant risks emerge only after systems are implemented and adapted within local workflows. This underscores the importance of *post-market surveillance*, continuous safety monitoring, and mechanisms for aggregating and learning from incident data across organisations. National and international guidance increasingly reflects this shift, emphasising learning systems rather than static certification as the foundation for digital safety (1, 55, 59, 62).

At the system level, incident-reporting and investigation structures play a critical role in making sociotechnical risks visible. However, to support meaningful learning, these systems must be equipped to capture interdependencies among system components, rather than narrowly focusing on proximate causes. The synthesis of findings across investigations, such as national thematic reviews, demonstrates the value of aggregating insights to identify recurrent patterns of risk that may not be apparent within single organisations (1, 63, 64).

Finally, the theory suggests that policy initiatives aimed at improving digital safety should prioritise transparency, shared accountability, and feedback loops between frontline users, organisations, and system designers. By supporting learning across system boundaries, policy and governance structures can help mitigate the propagation of recurring digital risks and contribute to the development of safer, more resilient digital healthcare systems (1, 24).

## Conclusion

As healthcare systems become increasingly dependent on digital technologies, understanding how health information technology–related risks arise, persist, and evolve in practice is essential for improving patient safety. This article has developed a sociotechnical theory of HIT-related risk that moves beyond explanations based on isolated technical failures or individual error, situating risk within the interactions among technology, work practices, organisational structures, and governance arrangements.

By conceptualising HIT-related risk as a dynamic process of emergence, concealment, propagation, and recoverability, the theory helps explain why many digital safety problems remain difficult to detect and resistant to traditional improvement strategies. It highlights how automation, information fragmentation, and adaptive workarounds can obscure vulnerabilities, while tight coupling and shared digital dependencies enable risks to scale over time, across settings, and among patients. Importantly, the theory also emphasises that safety in digital healthcare depends not only on preventing failure but on the capacity of organisations and systems to detect, contain, and learn from emerging hazards.

The contribution of this work is primarily theoretical. It provides an integrative sociotechnical framework that can support future empirical research, inform methodological choices in patient safety studies, and guide reflective practice in the design, implementation, and governance of digital health systems. As digital transformation continues to reshape healthcare delivery, such theory-informed perspectives will be increasingly necessary to support the development of safer, more resilient digital healthcare systems.

## Data Availability

The original contributions presented in the study are included in the article/Supplementary Material, further inquiries can be directed to the corresponding author.
